# CircRNA-ENO1 promoted glycolysis and tumor progression in lung adenocarcinoma through upregulating its host gene ENO1

**DOI:** 10.1038/s41419-019-2127-7

**Published:** 2019-11-25

**Authors:** Jiayu Zhou, Shizhen Zhang, Zhoumiao Chen, Zhengfu He, Yong Xu, Zhijun Li

**Affiliations:** 1 Department of Thoracic Surgery, Sir Run Run Shaw Hospital, School of Medicine, Zhejiang University, Hangzhou City, 310000 Zhejiang Province China; 2 Institute of translational Medicine, Zhejiang University, Hangzhou City, 310000 Zhejiang province China

**Keywords:** Non-small-cell lung cancer, Cell biology

## Abstract

Lung adenocarcinoma (LUAD) has long been one of the predominant reasons for the global cancer-linked mortality. The tumor progression is shown by several studies to be promoted by increased glycolysis. Enolase 1 (ENO1), as a glycolysis enzyme, performs pivotal role in glucose metabolism and contributes to tumor progression of numerous cancers. Circular RNAs (circRNAs) are catching increasing attentions for their surging roles in regulating gene expression in cancers. Our work is to uncover the regulatory mechanism circ-ENO1 on its host gene ENO1 and its function in glycolysis and tumor progression. Circ-ENO1 and its host gene ENO1 were identified to be upregulated in LUAD cells. Functionally, silencing circ-ENO1 retarded glycolysis, inhibited proliferation, migration and EMT, induced apoptosis. The cytoplasmic localization of circ-ENO1 was determined by FISH and subcellular fractionation. Mechanistically, circ-ENO1 acted as a ceRNA to interact with miR-22-3p and upregulate ENO1 expression. In vivo experiments certified that circ-ENO1 drove tumor growth and metastasis in vivo. In summary, current study elucidated that circ-ENO1 promoted glycolysis and tumor progression in LUAD by miR-22-3p/ENO1 axis, indicating circ-ENO1 as a promising treatment target for LUAD patients.

## Introduction

Lung cancer is known to be a major contributor of global tumor-related deaths, the worldwide 5-year survival rate of which is around 16.6%^1,2^. Lung adenocarcinoma (LUAD), a common subtype of lung cancer, takes up 30–35% of the primary lung cancers^3^. Although recent years have witnessed the advancement of clinical and experimental oncology for lung cancer, the prognosis of LUAD patients sees no dramatic rise^4^. Hence, improving the understanding of mechanisms behind tumor progression and tumor metastasis in lung cancer is imminently required.

Circular RNAs (circRNAs) are generated through exon skipping or back-splicing without either 5′-3′ polarity or the polyadenylated tail^5,6^. Attentions on circRNAs rose since they have been discovered as post-transcriptional modulators for gene expression. CircRNAs can function through competing endogenous RNA (ceRNAs) network, competitively targeting certain miRNAs to upregulate mRNAs^7,8^. The roles of circRNAs in promoting tumor progression have been largely revealed in a diversity of cancers^9–11^, including lung cancer^11,12^. We identified a new circRNA, circ-0000013, upregulated in LUAD through circRNA sequencing. To date, no study has explored circ-0000013 in cancers yet.

Glycolysis, also known as Warburg effect, refers to the transformation of glucose into lactate in cancer cells under the aerobic conditions^13^. During this glucose metabolism, large quantities of lipids, proteins, and nucleotides are produced, which helps accelerating the proliferation and division of cancer cells^14,15^. Increasing studies have unveiled the significance of glycolysis in tumor progression of lung cancer^16,17^. Enolase1 (ENO1) is a glycolytic enzyme. By conversing 2-phosphoglycerate into phosphoenolpyruvate, ENO1 performs crucial roles in aerobic glycolysis, and acts as a key contributor to Warburg effect in cancer cells^18,19^. Emerging studies have documented that ENO1 promotes tumor progression of lung cancer^20^. For example, ENO1 is proved to accelerate glycolysis, proliferation, migration, and invasion in non-small cell lung cancer via PI3K/AKT pathway^21^. Present study discovered that ENO1 was a host gene for circ-0000013 through circBase, so we renamed circ-0000013 as circ-ENO1. However, the regulation of ENO1 by circ-ENO1 has never been explored before.

Therefore, our study was attempted to investigate how circ-ENO1 functioned in LUAD, and how it regulated ENO1 and glycolysis.

## Materials and methods

### Tissue collection

Sixty-four pairs of LUAD tissues and the matched adjacent normal tissues were obtained from Sir Run Run Shaw Hospital, School of Medicine, Zhejiang University, with all patients signed the informed consents. The patients had undergone no other chemo- or radio-therapies before surgery. This study was approved by the ethics committee of Sir Run Run Shaw Hospital, School of Medicine, Zhejiang University. The tissues were stored immediately at −80 °C in nitrogen for later use.

### Cell lines and cell culture

The human LUAD cell lines including SPCA1, H1299, H1975 and A549 were used in this study. SPCA1 cells were provided by Cell Bank of Chinese Academy of Sciences, whereas the others were provided by Type Culture Collection of the Chinese Academy of Sciences (Shanghai, China). And 16HBE (the normal human bronchial epithelial cell line), and HEK-293T (the human embryonic kidney 293T cell lines) were also from Type Culture Collection of the Chinese Academy of Sciences (Shanghai, People’s Republic of China). All cells mentioned above were cultivated applying RPMI-1640 medium (Gibco, life technologies, California, USA). The mediums were added with 10% fetal bovine serum and 100 mg/mL streptomycin plus 100UI/mL of penicillin (Gibco, life technologies, California, USA). The incubation atmosphere was at 37 °C and contained 5% CO_2_.

### Cell transfection

In order to overexpress ENO1 or circ-ENO1, the pcDNA3.1 or pcDNA3.1(+) vectors (Invitrogen) were inserted with the sequences of ENO1. For the knockdown of circ-ENO1 or ENO1, small interfering RNAs specifically targeting circ-ENO1 (si-circ-ENO1#1, si-circ-ENO1#2, and si-circ-ENO1#3), or targeting ENO1 (si-ENO1#1, si-ENO1#2, and si-ENO1#3) were applied. The negative controls were empty vector (pcDNA3.1 or pcDNA3.1(+)) or scramble siRNAs (siNC). MiR-22-3p was overexpressed by miR-22-3p mimic and silenced by miR-22-3p inhibitor, with NC mimic and NC inhibitor (GenePharma, Shanghai, China) as control. SPCA1 and A549 cells were seeded onto the 24-well plates at an approximate density of 5 × 10^4^ cells per well. The transfection was finished by Lipofectamine 3000 (Invitrogen). RT-qPCR analysis was performed to evaluate the transfection efficiency. Subsequent to the 48-h cultivation, cells were subjected to function assays. The siRNA sequences were:

si-circ-ENO1#1: GGCUUCUGUAGAAGUUCUAAG (sense)

UAGAACUUCUACAGAAGCCAA(antisense);

si-circ-ENO1#2: CUGUAGAAGUUCUAAGGAAGC (sense)

UUCCUUAGAACUUCUACAGAA(antisense);

si-circ-ENO1#3: GCUUCUGUAGAAGUUCUAAGG (sense)

UUAGAACUUCUACAGAAGCCA(antisense);

si-ENO1#1: GCUGCUUACUGUAACUGUAUC(sense)

UACAGUUACAGUAAGCAGCUG (antisense);

si-ENO1#2: GGAGUUGGAGACCAGUCUAGC (sense)

UAGACUGGUCUCCAACUCCUG (antisense);

si-ENO1#3: GAGUGGUUUGCUUAGUCAAUG (sense)

UUGACUAAGCAAACCACUCUU(antisense).

### Lentivirus production and infection

The sh-circ-ENO1 sequences were inserted into pFH-L vectors (Shanghai Hollybio, China) containing green fluorescent protein (GFP) which acted as a detectable marker. Then, reconstructed vectors were introduced into HEK293T cells for the generation of lentiviruses, together with the pCMVΔR8.92 and the pVSVG-I as packing vectors (Shanghai Hollybio, China). Ninety-six hours following transfection, supernatants, which contained the lentivirus expressing sh-circ-ENO1 or the sh-NC (lv-sh-circ-ENO1 or lv-sh-NC) were harvested. SPCA1 and A549 cells then underwent the treatment of lv-sh-circ-ENO1 for 96 h (multiplicity of infection, 50). The infection efficiency was evaluated by examining the ratio of GFP positive cells under the fluorescence microscope. The SPCA1 and A549 cells transfected with lv-sh-circ-ENO1 were then subjected to rescue assays.

### Quantitative real-time polymerase chain reaction (RT-qPCR)

RNAs were obtained from indicated cells with the aid of Trizol (Invitrogen) adding DNase I. RNA was converted into cDNA applying the SuperScript III® (Invitrogen). The generate cDNA underwent amplification using real-time polymerase chain reaction (RT-qPCR) referring to TaqMan method with the BioRad CFX96 Sequence Detection System (BioRad company, Berkeley, CA). The levels of circRNAs and mRNAs were normalized to the levels of GAPDH, whereas the levels of miR-22-3p were normalized to U6. Results of RT-qPCR were evaluated and presented relative to the threshold cycle value (CT value), and were converted into the fold change.

### Cell counting Kit 8 assay

Cell viability was tested by Cell Counting Kit-8 (CCK-8, Corning Corporation, USA). In short, SPCA1 and A549 cells were plated into the 6-well plate with 1 × 10^3^ cells in each well with the media with the supplementation of 10% FBS. Optical density value (OD value) at 450 nm was determined 2 h subsequent to the addition of CCK-8 with the utilization of the Synergy 2 microplate reader (BioTek Instruments, US) at day 1, 2, 3, and 4. In addition, the evaluation of the influence of CoCl_2_ on cell viability was carried out as previous description^22^. And SPCA1 and A549 cells were treated with CoCl2 at a dose of 0, 50, 100, 200, 300, and 400 μmol/L.

### Enhanced ATP assay

The preparation of samples was conducted referring to the guidance of Enhanced ATP assay kit (Beyotime, Shanghai, China). The supernatants of each sample (20 μl) were added to the ATP detection solution (100 μl) attaching to the kit. Then, Promega Glomax 20/20 luminometer (Madison, WI, USA) was utilized to record the RLU values. The protraction of standard curve was conducted on the basis of the RLU values of ATP with the concentration of 0, 0.01, 0.05, 0.1, 0.5, 1, 5, and 10 nmol/L. Finally, the protein concentration was used to standardize the results, which were presented as ATP/protein (nmol/mg).

### Enolase activity

Enolase activity was detected utilizing the Enolase Activity Assay Kit (Sigma, Germany, MAK178-1KT). Briefly, the lysates of A549 and SPCA1 cells were mixed with the reaction buffer and subjected to incubation at 25 °C. Subsequent to 5–10 min, the initial examination of OD value was carried out at the wavelength of 570 nm, followed by the examination 2–3 min a time until the OD value in the most active sample went beyond highest standard. The calculation of enolase activity was conducted using the equation described previously^23^.

### Measurement of glucose and lactate

Forty-eight hours after transfection, A549 and SPCA1 cells were cultured in the phenol red free medium for 1 day, and then the glucose uptake and production of lactate were examined utilizing the Glucose Assay Kit and Lactic Acid Kit (Jiancheng Bioengineering Institute, China).

### 5-Ethynyl-2′-deoxyuridine (EdU) staining

Cell proliferative capacity was tested with the aid of a Cell-Light EdU DNA Cell Proliferation Kit (RiboBio Co., Ltd, Guangzhou, China). Cells were plated in 96-well plates in the concentration of 5 × 10^3^ cells each well. Subsequent to the incubation of 12 h, the cells went through fixation in 4% formaldehyde and then 30-min incubation in 1 × Apollo® reaction buffer (100 μl). DAPI was utilized for staining the cell nuclei. The fluorescence of EdU or DAPI were viewed utilizing a fluorescence microscopy (Nikon, Tokyo, Japan). IMAGEJ software was used for the calculation of the EdU-positive cells.

### Transferase-mediated dUTP nick end labeling staining

Transferase-mediated dUTP nick end labeling (TUNEL) assay was implemented to determine cell apoptosis by utilizing the detection kit (Roche, Mannheim, Germany). The cells with nuclei which was stained brown were considered to be TUNEL-positive cells. The apoptotic cells were calculated in five randomly selected visual fields of each slice. Apoptotic ratio was calculated based on the following formula: (number of positive cells/number of all counted cells) × 100%.

### Transwell migration assay

The transwell migration assay was conducted utilizing the 24-well transwell chambers with 8.0-μm–pore polycarbonate filter inserts (Costar, San Diego, CA, USA). Shortly, cells at 1 × 10^5^ cells per well were re-suspended using the non-serum medium (500 µl) and were put in the upper compartment. And the lower compartment was with the supplementation of 10% FBS-contained DMEM (500 µl). In subsequence to 48 h, the migrated cells in lower compartment underwent 10 min fixation in 4% paraformaldehyde and then 30 min staining utilizing 1% crystal violet at room temperature. The counting of invaded cells was accomplished applying a microscope.

### Wound healing assay

The migratory capacity of cells was tested by wound healing assay. All cells were cultivated in the six-well plates. A day after, the 10 μl pipette was used to scratch a wound at the middle of each well. Then, the medium was substituted with the 1% FBS-contained fresh medium. Twenty-four hours after the capture of the first picture of the wound, the second picture of the wound was obtained. The percentage of migration was evaluated utilizing Image Pro Plus.

### Nuclear-cytoplasmic fractionation

The cytoplasm and nucleus of SPCA1 and A549 cells were separated with the aid of a Nuclear and Cytoplasmic Extraction Reagents (Thermo Scientific, USA). The analysis of RNA was conducted by RT-qPCR. The markers for nucleus and cytoplasm were respectively U6 and GAPDH.

### Immunofluorescence

Total cells on the slides went through permeabilization in 0.3% Triton X-100 subsequent to the fixation in 4% paraformaldehyde. After that, goat serum was utilized for the blocking. Cells were later cultivated overnight with anti-E-cadherin (ab40772, Abcam), and anti-N-cadherin (ab76057, Abcam) at 4 °C. Then, slide was subjected to 1-h incubation with the anti-rabbit Alexa Fluor 488 (Jackson Immunoresearch, West Grove, PA, USA) under the room temperature. The counterstaining of nucleus was accomplished by using DAPI. Each sample was viewed with the use of the fluorescence microscope (DMI4000B, Leica). The counting of positive cells was conducted randomly in the ×40 magnification.

### Fluorescence in situ hybridization analysis (FISH)

The separation of cytosolic and nuclear fractions was conducted with the aid of a PARIS kit (Life Technologies). The probes for RNA Fluorescence in situ hybridization analysis (FISH) targeting circ-ENO1 were designed and produced by Bogu referring to the instruction of manufacturer. After the fixation in 4% formaldehyde and then PBS washing, cells were subjected to pepsin treatment and dehydration using ethanol. Then the dried cells were cultivated with FISH probe (40 nM) in the hybridization buffer. Subsequent to hybridization and washing, the slide was dehydrated and was later mounted using Prolong Gold Antifade Reagent as well as DAPI for further detection. A fluorescence microscopy (DMI4000B, Leica) was used to view the immunofluorescence of each slide.

### Luciferase reporter assay

To carry out luciferase reporter assay, luciferase reporter gene-encoded pGL3 plasmids were provided by Promega Corporation (Madison). The plasmid with circ-ENO1 or ENO1 3’UTR containing the wide type or mutant sides for miR-22-3p, namely WT-circ-ENO1, Mut-circ-ENO1, WT-ENO1, and Mut-ENO1, were constructed. Then, WT-circ-ENO1, Mut-circ-ENO1, WT-ENO1, or Mut-ENO1 were co-transfected with miR-22-3p mimic or NC mimic into HEK-293T cells applying the Lipofectamine 2000 (Invitrogen). Also, to detect the effect of circ-ENO1 on the promoter transcription of ENO1, ENO1 promoter reporter plasmids were transfected with si-NC, si-circ-ENO1#1 or ci-circ-ENO1#2 into HEK-293T cells applying the Lipofectamine 2000 (Invitrogen). Cells were harvested following the transfection for 48 h, and analyzed by utilizing the Dual-Luciferase Reporter Assay system (Promega), with Renilla luciferase activity as normalized control.

### RNA immunoprecipitation (RIP) assay

A549 and SPCA1 cells were scraped in the IP lysis buffer and then mechanically separated utilizing the homogenizer. The cell lysates were added with antibodies against Ago2 and were subjected to overnight incubation at 4 °C. After the addition of streptavidin-coated magnetic beads and 2 h incubation, the beads underwent resuspension using 1 mL TRIzol. cDNA was later produced from the isolated RNA, and subjected to RT-qPCR analysis.

### Western blot

The harvested cells were digested by RIPA buffer. Following sonication, the samples were centrifuged for 15 min at 12,000 g under 4 °C. For the determination of total protein density, a DC Protein Assay Kit I (Bio-Rad, Richmond, CA, USA) was applied. Then, after separating the proteins were on 12% SDS-PAGE, transfer the proteins onto the hybond nitrocellulose membrane (Amersham, NJ, USA). In all, 5% nonfat milk was utilized for the sealing of membranes in the Tris-buffered saline (pH 7.5). The membrane went through overnight hybridization with the primary antibodies, and then second antibodies. The protein bands were revealed by an ECL kit (Millipore, Billerica, MA, USA). The expression levels of proteins expression levels were evaluated by the Image J (National Institutes of Health, USA). The primary antibodies were: anti-ENO1 (ab155102) anti-E-cadherin (ab1416), anti-N-cadherin (ab18203), anti-PARP (ab74290), anti-cleaved-caspase 3 (ab2302), caspase-3 (ab13847), anti-cleaved-caspase 6 (ab2326), anti-caspase 6 (ab185645), anti-cleaved-caspase 9 (ab2324), anti-caspase 9 (ab52298), and anti-GAPDH (ab181602) all from Abcam (Cambridge, UK).

### Tumor xenograft

The BALB/C-nu mice with 5-week old were caged and kept in the laminar airflow cabinets in the specific pathogen-free environment. A549 cells transfected with lv-sh-circ-ENO1 or lv-sh-NC were re-suspended in PBS. Then the lv-sh-circ-ENO1 or sh-NC transfected A549 cells were respectively inoculated subcutaneously into the BALB/C-nu mice at a density of 1 × 10^7^ cells each mouse with 200 μl PBS, with six mice in each group. One week after injection, the mice were tested three times a week for a total of 3 weeks. The growth of xenografts was assessed by determining the width and length of tumor mass.

### Immunohistochemistry (IHC)

The tissue blocks were paraffin-embedded and sliced into 4 μm slides. The antibodies against Ki67 (ab15580), ENO1 (ab155102), E-cadherin (ab1416), and N-cadherin (ab98952) from Abcam (Cambridge, UK) were used. Immunohistochemistry (IHC) analysis was performed according previous descriptions^24^.

### In vivo metastasis

A549 cells stably transfected with sh-NC or sh-circ-ENO1 were transplanted into the nude mice via tail vein injection. Five mice in each group. 8 weeks later, the mice were killed for the collection of lung. The metastatic nodules were calculated after staining and IHC analysis.

### Statistical analysis

Data computation was accomplished by SPSS software 16.0 (SPSS Inc., Chicago, IL, USA). For determining the significance of differences between two groups or among multiple groups, Student’s *t*-test or one-way ANOVA was applied. Each experiment was conducted for three times at minimum. The statistical significance in differences were confirmed when *p* < 0.05.

## Results

### Circ-ENO1 was a bona fide circRNA upregulated in LUAD tissues and cells

**Fig. 1:**
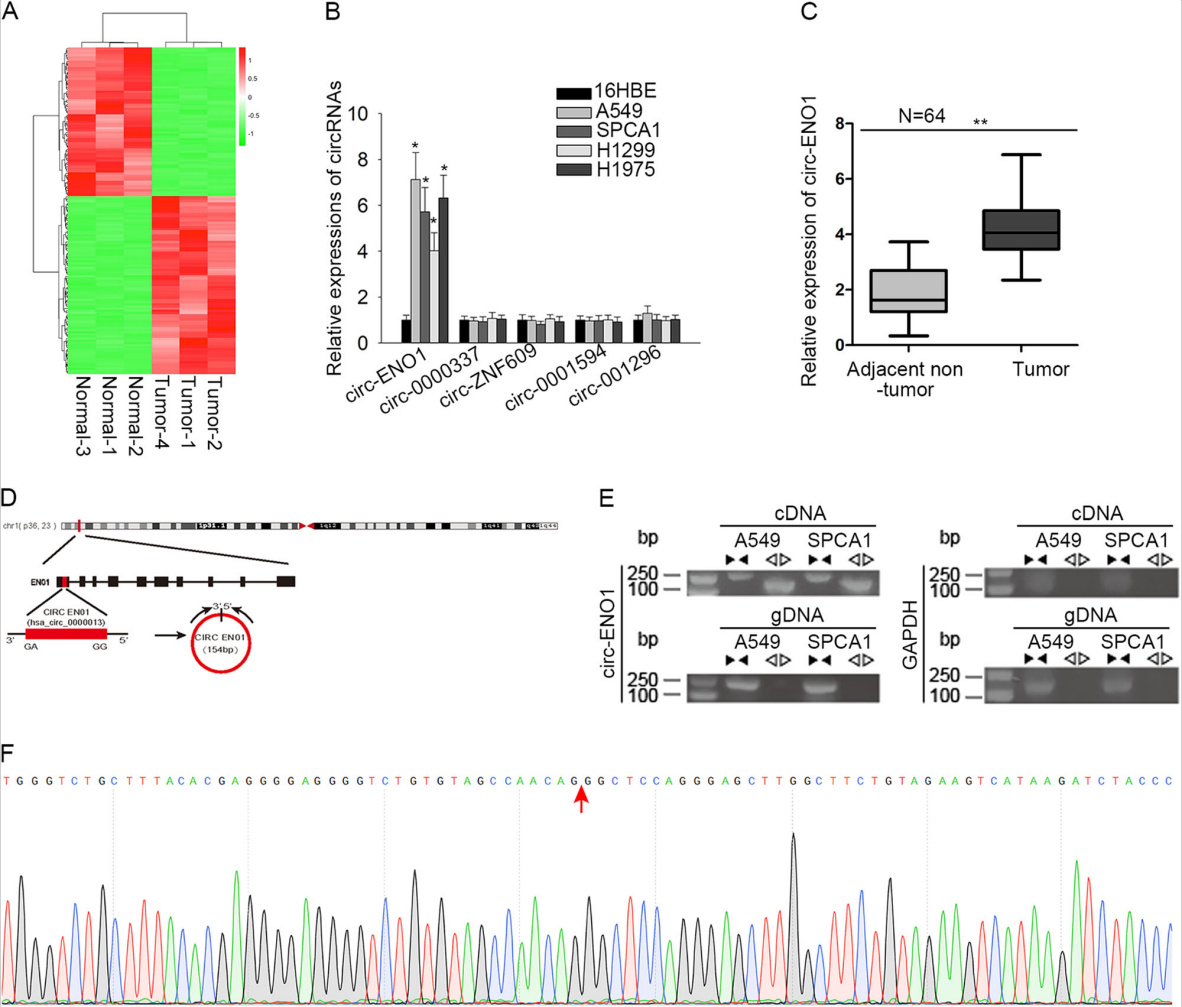
Circ-ENO1 and its host gene ENO1 were upregulated in lung cancer cells. **a** Hierarchical clustering showed the differentially expressed circRNAs in three LUAD tissues compared with the paired normal ones by circRNA sequencing, and the top 5 upregulated cricRNAs (circ-ENO1, circ-0000337, circ-ZNF609, circ-0001594, and circ-001296) were selected for further detection. **b** Expressions of circ-ENO1, circ-0000337, circ-ZNF609, circ-0001594, and circ-001296 were detected by RT-qPCR in lung cancer cells (A549, SPCA1, H1299, and H1975) and normal cells (16HBE). **c** circ-ENO1 expression was measured in 64 pairs of LUAD tissues and normal lung tissues. **d** The genomic location of circ-ENO1. **e** The agarose gel electrophoresis which showed that circ-ENO1 amplified by divergent primers was detected only in cDNA. **f** The sequencing results of circ-ENO1. ^*^p < 0.05.

First, to find out the circRNA potentially regulating ENO1, we carried out circRNA sequencing. The hierarchical clustering showed that differentially expressed circRNAs in three LUAD tissues compared with the paired normal tissues (Fig. 1a). We then selected the top 5 upregulated circRNAs (circ-ENO1, circ-0000337, circ-ZNF609, circ-0001594, and circ-001296) and detected their expressions in cell lines. Interestingly, only circ-ENO1 was upregulated in in four LUAD cell lines (A549, SPCA1, H1299, and H1975) and the normal cells (16HBE) (Fig. 1b). Then, the expression of circ-ENO1 was higher in LUAD tissues than that in normal lung tissues (Fig. 1c). Circular structure of circ-ENO1 was illustrated in Fig. 1d. Moreover, we revealed through PCR that circ-ENO1 amplified by divergent primers was detectable in cDNA rather than genomic DNA (gDNA), which confirmed that circ-ENO1 was a bona fide circRNA (Fig. 1e). Additionally, we figured out through circRNA sequencing that circ-ENO1 was 154 bp in length and was back spliced from ENO1 gene (Fig. 1f). These results suggested that circ-ENO1 was a bone fide circRNA upregulated in LUAD.

### Silencing circ-ENO1 prohibited proliferation and facilitated apoptosis in LUAD cells

**Fig. 2:**
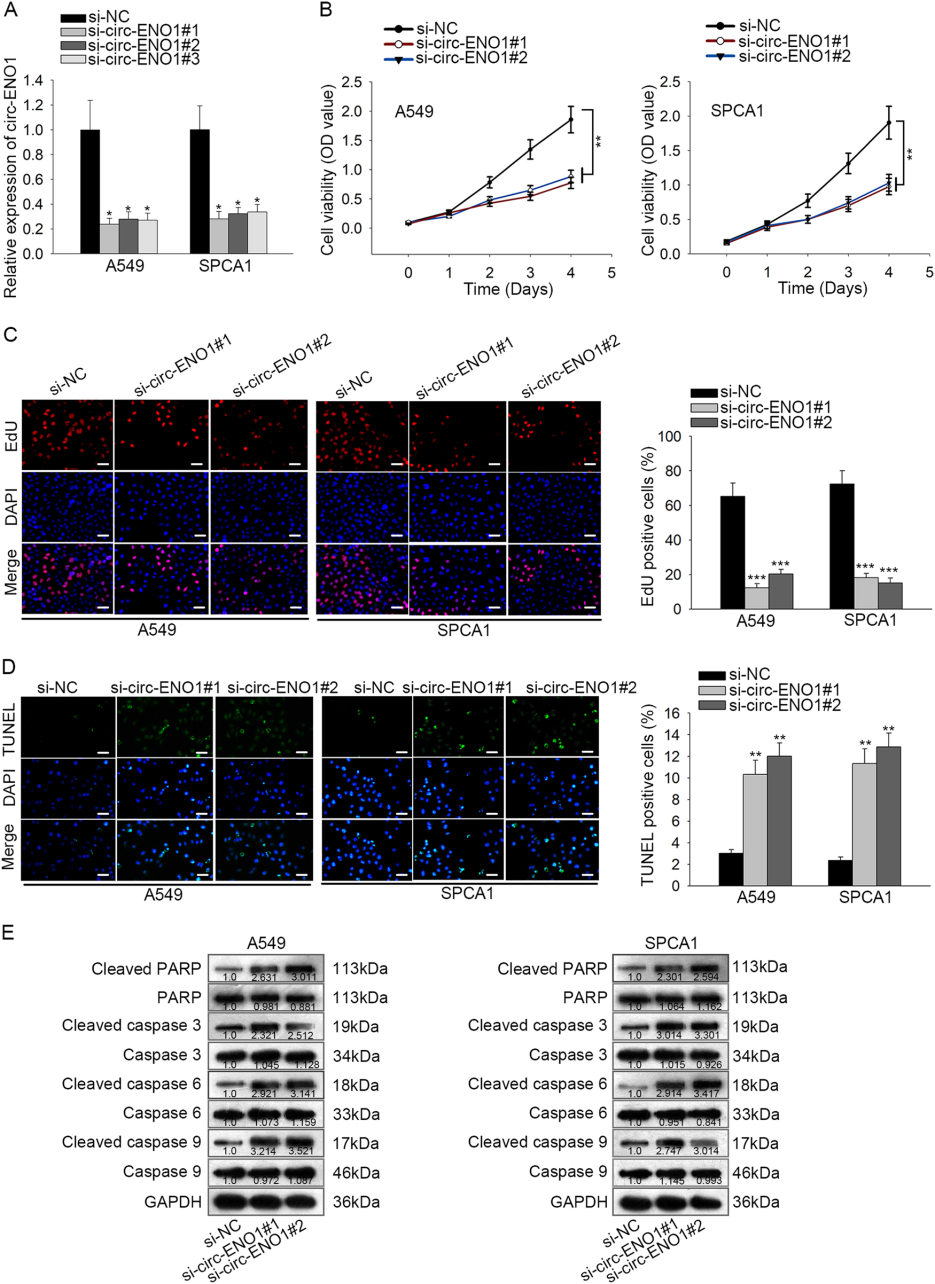
Silencing circ-ENO1 prohibited proliferation and facilitated apoptosis in LUAD cells. **a** Knockdown efficiency of circ-ENO1 by si-circ-ENO1#1, si-circ-ENO1#2, and circ-ENO1#3 in A549 and SPCA1 cells was determined by RT-qPCR analysis, with si-NC as negative control. **b**, **c** A549 and SPCA1 cells were transfected with si-NC, si-circ-ENO1#1, or si-circ-ENO1#2 for subsequent assays. CCK-8 and EdU assays were used to detect cell proliferation in each group. **d** TUNEL assay was used to detect cell apoptosis in each group. **e** Western blot assay was used to detect the expression levels of cleaved PARP, PARP, cleaved-caspase 3, caspase 3, cleaved-caspase 6, caspase 6, cleaved-caspase 9, and caspase 9, with GAPDH as normalized control. ^*^p < 0.05, ^**^p < 0.01, ^***^p < 0.001.

To assess the function of circ-ENO1 in LUAD, we designed loss-of-function assays by silencing circ-ENO1 in A549 and SPCA1 cells via si-circ-ENO1#1/2/3. Results of RT-qPCR showed that si-circENO1#1 and si-circ-ENO1#2 presented the best knockdown efficiency (Fig. 2a). Therefore, we used si-circENO1#1 and si-circ-ENO1#2 for subsequent experiments. CCK-8 and EdU results showed that silencing circ-ENO1 retarded proliferation of both A549 and SPCA1 cells (Fig. 2b, c). TUNEL assay showed that the staining ratio of TUNEL was increased upon circ-ENO1 silencing, indicating that circ-ENO1 knockdown facilitated apoptosis of LUAD cells (Fig. 2d). Additionally, we detected the expressions of apoptosis-related genes by western blot upon circ-ENO1 silencing, and found that the level of PARP decreased, whereas the levels of cleaved-caspase 3, cleaved-caspase 6 and cleaved-caspase 9 were increased by circ-ENO1 depletion (Fig. 2e). Together, results above indicated that silencing circ-ENO1 prohibited proliferation and facilitated apoptosis in LUAD cells.

### Silencing circ-ENO1 retarded migration and EMT in LUAD cells

**Fig. 3:**
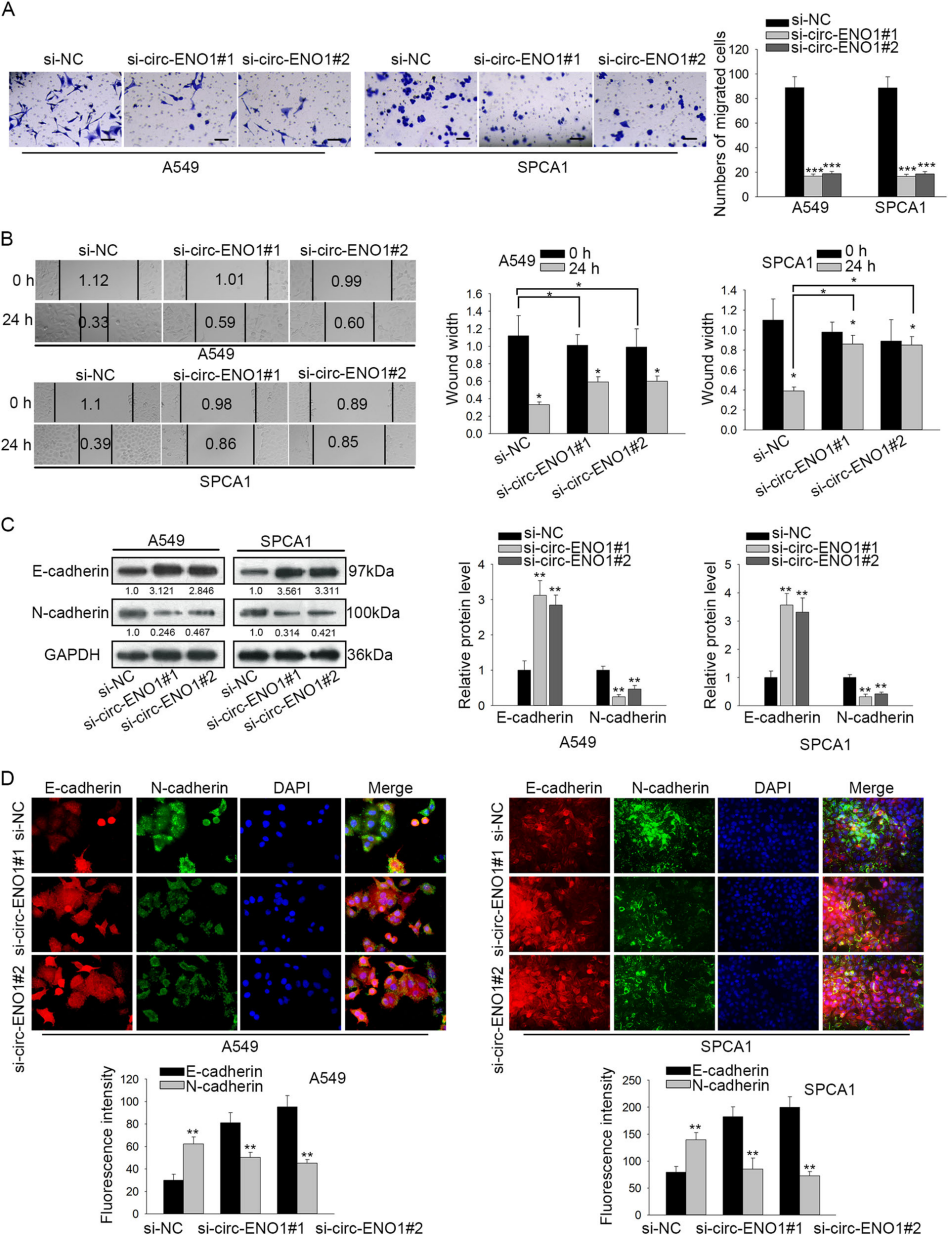
Silencing circ-ENO1 retarded migration and EMT in LUAD cells. **a**, **b** Cell migration was determined by transwell assay and wound healing assay. **c**, **d** The expressions of E-cadherin and N-cadherin were detected by western blot and IF assays. ^*^p < 0.05, ^***^p < 0.001.

Furthermore, we detected the effect of circ-ENO1 on migration and EMT. Results of transwell migration assay and scratch wound assay demonstrated that the knockdown of circ-ENO1 weakened the migratory ability of A549 and SPCA1 cells (Fig. 3a, b). Western blot data illustrated that the level of epithelial marker E-cadherin was increased whereas the level of mesenchymal marker N-cadherin was decreased in response to circ-ENO1 silencing (Fig. 3c). Same results were also observed from immunofluorescence assay (Fig. 3d). Collectively, these results suggested that silencing circ-ENO1 retarded migration and EMT in LUAD cells.

### Silencing circ-ENO1 attenuated glycolysis through ENO1 in LUAD cells

**Fig. 4:**
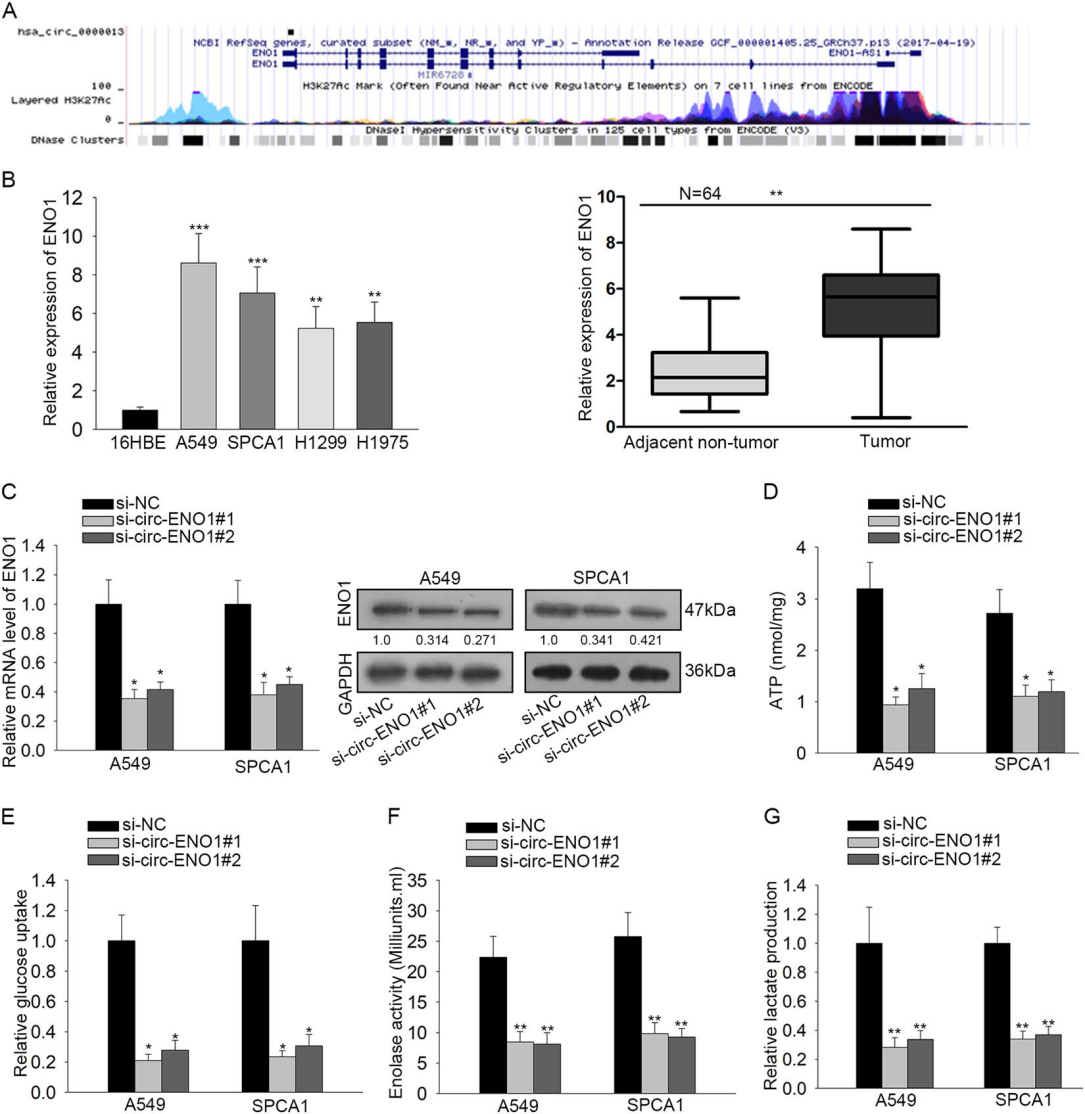
Silencing circ-ENO1 attenuated glycolysis through ENO1 in LUAD cells. **a** ENO1 was identified as the host gene of circ-ENO1 by browsing circBase. **b** RT-qPCR analysis showed the upregulation of ENO1 in LUAD cell lines and tissues. **c** ENO1 expression was detected by RT-qPCR and western blot analyses under circ-ENO1 silencing. **d**–**g** The levels of ATP, glucose uptake, enolase activity, and lactate production upon circ-ENO1 silencing. ^*^p < 0.05, ^**^p < 0.01, ^***^p < 0.001.

It is axiomatically known that circRNAs usually exert their functions through regulating certain gene expressions. Herein, to find out the target gene for circ-ENO1, we browsed circBase (http://www.circbase.org/). We found that ENO1 was the host gene of circ-ENO1 (Fig. 4a), indicating the regulatory potential of circ-ENO1 on ENO1. Hence, we focused on the investigation of ENO1. We found by RT-qPCR analysis that ENO1 exhibited higher expression in four LUAD cell lines and LUAD tissues (Fig. 4b).

Since ENO1 is reputed as a glycolytic enzyme playing key roles in Warburg effect in cancer cells^18,19^, and previous studies have demonstrated that ENO1 could facilitate glycolysis and promote tumor progression in lung cancer^21^, we tried to examine whether circ-ENO1 could regulate ENO1 expression and influence glycolysis in LUAD cells. Through RT-qPCR and western blot analysis, we found that silencing circ-ENO1 caused a reduction on the expression of ENO1 (Fig. 4c). It is acknowledged that reduced ENO1 activity would influence the level of glycolysis-related products theoretically^25^, so we examined the effect of circ-ENO1 on the glycolysis. It turned out that the depletion of circ-ENO1 reduced the level of ATP, retarded the relative glucose uptake, attenuated enolase activity, as well as decreased the lactate production (Fig. 4d–g). Taken together, it was implied that silencing circ-ENO1 attenuated glycolysis through ENO1 in LUAD cells.

### Circ-ENO1 upregulated ENO1 expression through sponging miR-22-3p in LUAD cells

**Fig. 5:**
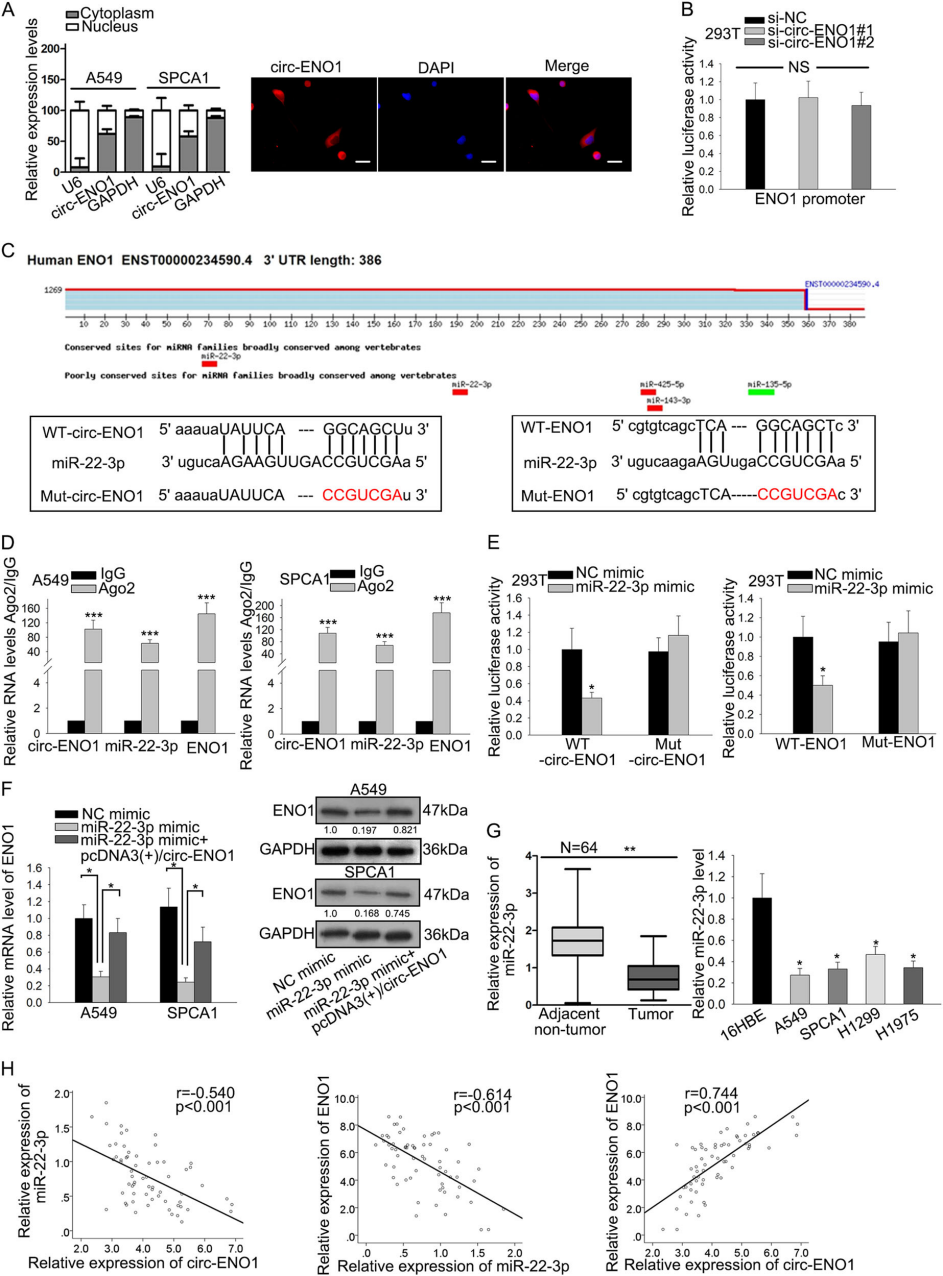
Circ-ENO1 upregulated ENO1 expression through sponging miR-22-3p in LUAD cells. **a** Subcellular fractionation and RNA FISH assay confirmed the cytoplasm localization of cric-ENO1. **b** Luciferase reporter assay showed the non-effect of circ-ENO1 silencing on ENO1 promoter transcription. **c** The binding sites and mutant sites on circ-ENO1 and ENO1 for miR-22-3p. **d**, **e** RIP and luciferase reporter assays validated the binding of miR-22-3p with circ-ENO1 and ENO1. **f** Results of RT-qPCR and western blot confirmed the effect of circ-ENO1/miR-22-3p axis on ENO1 expression. **g** RT-qPCR results showed the downregulation of miR-22-3p in LUAD tissues and cell lines. **h** Spearman’s correlation curve showed the negative correlation between miR-22-3p with circ-ENO1 and ENO1, and the positive correlation between circ-ENO1 and ENO1. ^*^p < 0.05, ^**^p < 0.01, ^***^p < 0.001.

Subsequently, we interrogated the modulatory mechanism of circ-ENO1 on ENO1 expression. By subcellular fractionation and RNA FISH analysis, we identified the expression of circ-ENO1 in cytoplasm (Fig. 5a). Through luciferase reporter assay, we discovered that silencing circ-ENO1 had no impact on the luciferase activity of ENO1 promoter reporter (Fig. 5b). These results indicated that circ-ENO1 might regulated ENO1 at post-transcriptional level. Considering that in cytoplasm, ceRNA mechanism has been documented as a classic way whereby circRNAs regulate gene expression^26^, including in LUAD^27^, we speculated that circ-ENO1 might also regulated ENO1 expression through this way. With the aid of bioinformatics tools including TargetScan (http://www.targetscan.org/vert_72/), we identified that ENO1 containing the conserved binding sites for only miR-22-3p (Fig. 5c), and then through browsing Starbase 3.0 (http://starbase.sysu.edu.cn/), we identified miR-22-3p was putative binding miRNA for circ-ENO1. Therefore, we chose miR-22-3p for further detection.

The binding sites and mutant sites of circ-ENO1 and ENO1 for miR-22-3p were presented in Fig. 5c. RIP assays confirmed that circ-ENO1, miR-22-3p and ENO1 could be immunoprecipitated by Ago2 antibody (Fig. 5d), indicating the interaction of miR-22-3p with circ-ENO1 and ENO1. Additionally, we overexpressed miR-22-3p to conduct luciferase reporter assays. The overexpression of miR-22-3p by miR-22-3p mimic was validated by RT-qPCR results (Supplementary figure 1A). Results of luciferase reporter assays showed that overexpression of miR-22-3p alleviated the luciferase activity of only WT-circ-ENO1 and WT-ENO1, but had no impact on the Mut-circ-ENO1 and Mut-ENO1 (Fig. 5e). Moreover, we tested the effect of circ-ENO1/miR-22-3p axis on ENO1 expression. The overexpression of circ-ENO1 by pcDNA3.1(+)/circ-ENO1 was determined by RT-qPCR (Supplementary figure 1B). RT-qPCR and western blot analyses depicted that overexpression of miR-22-3p reduced the mRNA and protein levels of ENO1, and co-expression of pcDNA3.1(+)/circ-ENO1 counteracted such effect (Fig. 5f).

We also detected the implication of miR-22-3p in LUAD. We observed that miR-22-3p exhibited significant downregulation in LUAD tissues and cell lines (Fig. 5g). Spearman’s correlation curve depicted that miR-22-3p had a negative correlation with circ-ENO1 and ENO1 expression in LUAD tissues, and that circ-ENO1 and ENO1 were positively correlated in LUAD tissues (Fig. 5h). These data suggested that circ-ENO1 upregulated ENO1 expression through sponging miR-22-3p in LUAD cells.

### Circ-ENO1/miR-22-3p/ENO1 regulated glycolysis so as to regulate proliferation, migration and EMT in LUAD cells

**Fig. 6:**
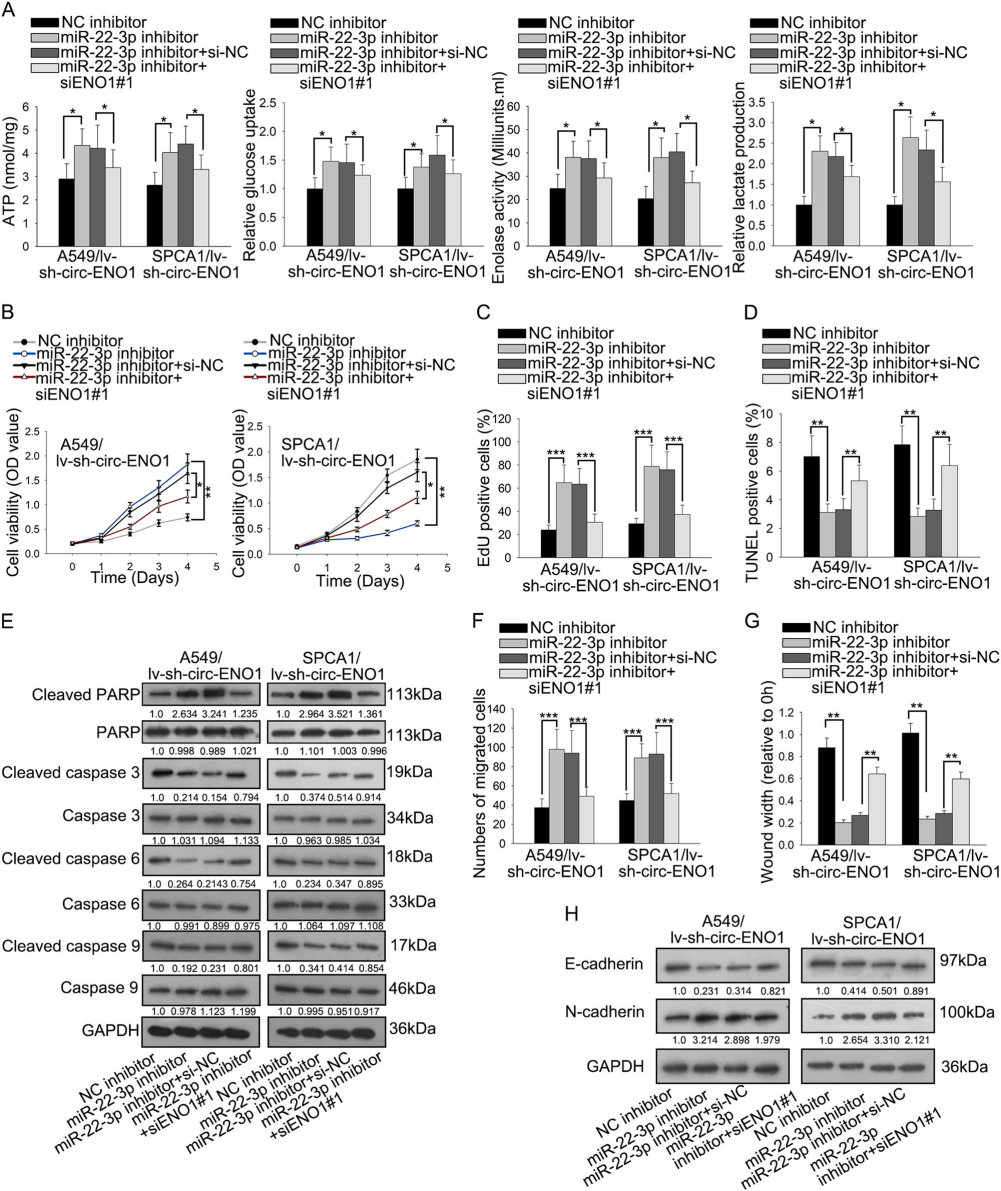
Circ-ENO1/miR-22-3p/ENO1 regulated glycolysis so as to regulate proliferation, migration and EMT in LUAD cells. A549 and SPCA1 cells were transfected with lv-sh-circ-ENO1. The lv-sh-circ-ENO1 transfected cells were transfected with NC inhibitor, miR-22-3p inhibitor, miR-22-3p inhibitor + si-NC, or miR-22-3p inhibitor + siENO1#1. **a** The levels of ATP, glucose uptake, enolase activity, and lactate production of each group. **b**, **c** CCK-8 and EdU assays were performed to detect proliferation of cells in each group. **d** TUNEL assay was used to detect cell apoptosis in each group. **e** Western blot assay was used to examine the expressions of PARP, cleaved-caspase 3, caspase 3, cleaved-caspase 6, caspase 6, cleaved-caspase 9, and caspase 9 in each group, with GAPDH as normalized control. **f**, **g** Transwell assay and wound healing assays were used to detect cell migration in each group. **h** Western blot assay was used to examine the expressions of E-cadherin and N-cadherin in each group. ^*^p < 0.05, ^**^p < 0.01, ^***^p < 0.001.

Later, we implemented rescue assays to examine the effect of circ-ENO1/miR-22-3p/ENO1 axis on glycolysis, proliferation, migration, and EMT in LUAD cells. A549 and SPCA1 cells were transfected with lv-sh-cric-ENO1 to stably silence circENO1 expression. Then, we inhibited miR-22-3p and silenced ENO1 in these transfected cells. The transfection of miR-22-3p inhibitor and si-ENO1#1/2/3 was confirmed by RT-qPCR results (Supplementary Fig. 1C). Since si-ENO1#1 exhibited the highest knockdown efficiency, we used it for subsequent assays. We found that inhibiting miR-22-3p facilitated the ATP level, glucose uptake, enolase activity, and lactate production in lv-sh-circ-ENO1 transfected A549 and SPCA1 cells, and these results could be counteracted by the co-transfection of si-ENO1#1 (Fig. 6a). Furtherly, miR-22-3p inhibition aggravated proliferation of lv-sh-circ-ENO1 transfected A549 and SPCA1 cells, and co-transfection of si-ENO1#1 reversed this results (Fig. 6b, c). Moreover, the prohibitive effect of silencing miR-22-3p on cell apoptosis could be countervailed by silencing ENO1 in lv-sh-circ-ENO1 transfected A549 and SPCA1 cells (Fig. 6d). Additionally, lv-sh-circ-ENO1 transfected A549 and SPCA1 cells with miR-22-3p knockdown presented elevated level of PARP and declined levels of cleaved-caspase3, cleaved-caspase 6, and cleaved-caspase 9, but co-transfection of si-ENO1#1 reversed such results (Fig. 6e). The migration ability of cells with lv-sh-circ-ENO1 transfection was increased by miR-22-3p depletion, which could be counteracted by silencing ENO1 (Fig. 6f, g). Western blot analyses revealed that in lv-sh-circ-ENO1-transfected LUAD cells, E-cadherin was decreased and N-cadherin was increased by miR-22-3p silencing, and that co-transfection of si-ENO1#1 renovated such effects (Fig. 6h). To sum up, results above indicated that circ-ENO1/miR-22-3p/ENO1 regulated glycolysis so as to regulate proliferation, migration and EMT in LUAD cells.

### Circ-ENO1 promoted tumor growth and metastasis in LUAD in vivo

**Fig. 7:**
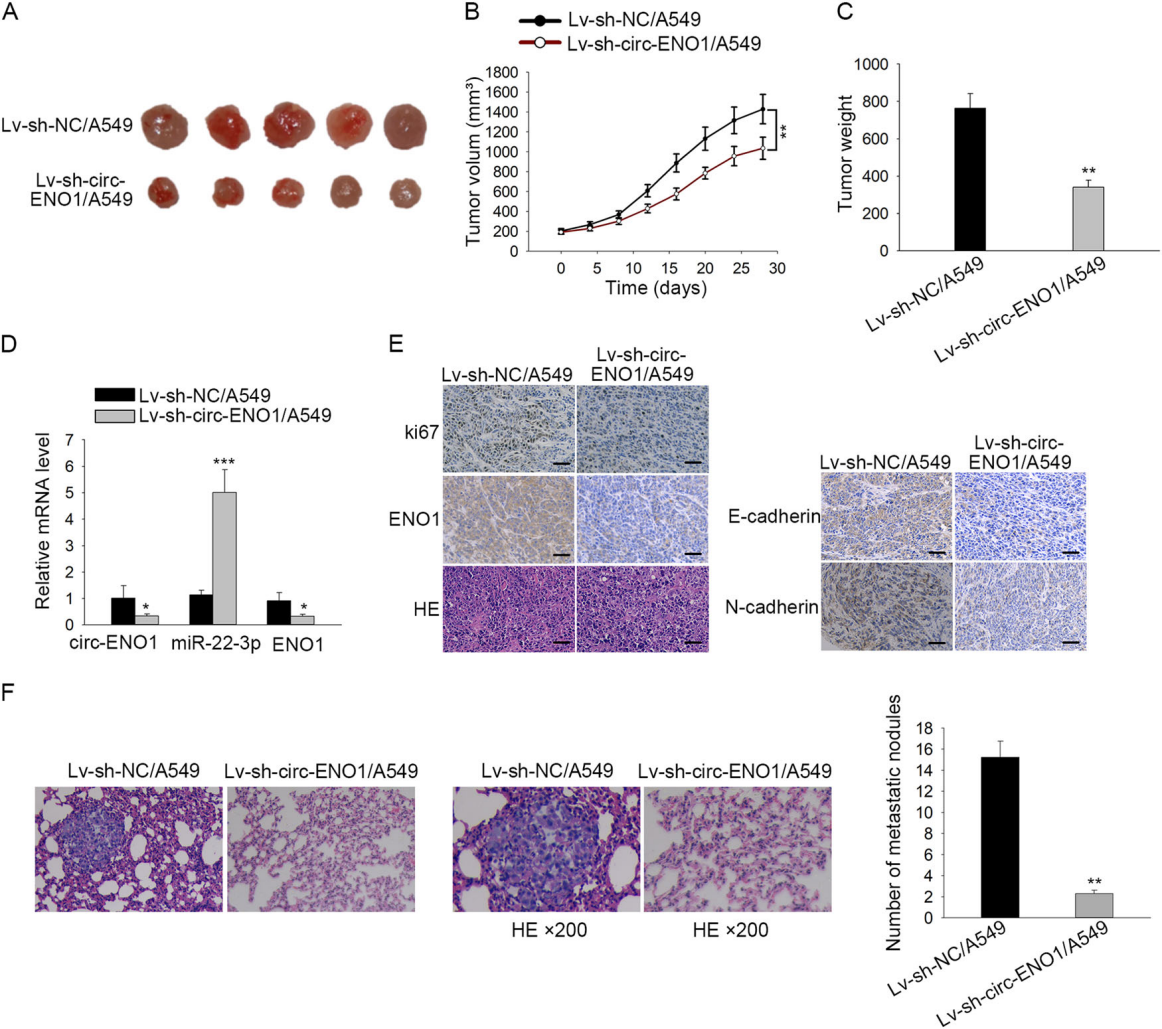
Circ-ENO1 promoted tumor growth and metastasis in LUAD in vivo. **a** The pictures of xenografts from mice injected with lv-sh-NC/A549 and lv-sh-circ-ENO1/A549. **b** The growth curve of xenograft mice injected with lv-sh-NC/A549 and lv-sh-circ-ENO1/A549. **c** The tumor weight from mice injected with lv-sh-NC/A549 and lv-sh-circ-ENO1/A549. **d** RT-qPCR results of the expressions of circ-ENO1, miR-22-3p and ENO1 in xenografts of mice injected with lv-sh-NC/A549 and lv-sh-circ-ENO1/A549. **e** IHC results of the positivity of Ki67, ENO1, E-cadherin, and N-cadherin of the xenografts from mice injected with lv-sh-NC/A549 and lv-sh-circ-ENO1/A549. **f** The pictures and HE staining results metastatic nodes of the xenografts from mice injected with lv-sh-NC/A549 and lv-sh-circ-ENO1/A549. ^*^p < 0.05, ^***^p < 0.001.

Finally, to further inquire the effect of circ-ENO1 on LUAD progression, in vivo assays were unfolded. A549 cells transfected with lv-sh-circ-ENO1 were injected into the right flanks of nude mice to generate xenografts. We discovered that lv-sh-circ-ENO1/A549 injected mice generated smaller tumors than control (Fig. 7a–c). RT-qPCR analyses confirmed that mice with circ-ENO1 silenced A549 cells presented lower expression of circ-ENO1 and ENO1, and higher expression of miR-22-3p (Fig. 7d). IHC results showed that the proliferation index Ki67 and ENO1 presented lower positivity upon circ-ENO1 silencing, and that E-cadherin presented higher positivity while N-cadherin presented lower positivity upon circ-ENO1 silence (Fig. 7e). We also observed that mice injected with lv-sh-circENO1/A549 had less metastatic nodes than control (Fig. 7f). Hence, results above indicated that circ-ENO1 promoted tumor growth and metastasis in LUAD in vivo.

## Discussion

LUAD is a common subtype of lung cancer with stagnant improvement in prognosjis during past decades despite of the treatment progress^3,4^. This indicates a call for the identification of new biomarkers by further molecular research on lung cancer.

Over the past decades, circRNAs have aroused increasing attentions since they are discovered as post-transcriptional modulators for gene expression. The roles of circRNAs in promoting tumor progression have been largely revealed in a diversity of cancers^28–31^, including lung cancer^32–34^. Herein, we identified through circRNA sequencing and RT-qPCR analysis that a new circRNA, circ-ENO1, was upregulated in both LUAD tissues and cell lines, indicating the participation of circ-ENO1 in LUAD. Functionally, our study demonstrated that silencing circ-ENO1 prohibited proliferation, induced apoptosis, facilitated migration and EMT in LUAD cells.

Glycolysis has been increasingly revealed as a hallmark for tumor progression in diversities of cancers, including in lung cancer. Induced glycolysis and increased glucose uptake under the aerobic conditions result in the facilitated production of lipids, proteins, and nucleotides, contributing to the proliferation and division of tumor cells^13–15^. Multiple genes related to glycolysis have been reported to participate in the cancer progression. ENO1 is a glycolysis enzyme responsible for the conversing 2-phosphoglycerate into phosphoenolpyruvate^35^. Previous studies have shown the involvement of ENO1 in tumor progression through regulating glycolysis in lung cancer^21^. In concordance, present study also identified the overexpression of ENO1 in LUAD tissues and cells. However, the detailed mechanism has never been explored before.

Through browsing circBase, we firstly discovered that ENO1 was the host gene for circ-ENO1, suggesting the regulatory potential of circ-ENO1 on ENO1 expression. Furthermore, we validated that silencing circ-ENO1 could reduce ENO1 expression and caused a decrease in ATP level, glucose uptake, enolase activity, and lactate production, indicating that circ-ENO1 regulated ENO1 expression to modulate glycolysis in LUAD cells.

The subcellular localization of circ-ENO1 was identified to be expressed mainly in cytoplasm, and the non-effect of silencing circ-ENO1 on ENO1 promoter transcription indicated the potential of circ-ENO1 to regulate ENO1 at post-transcriptional level. Previous studies have demonstrated that circRNAs can modulate gene expression through functioning as ceRNA^7,8^. In our study, we first identified through TargetScan that ENO1 contains conserved sites only for miRNA-22-3p. Former study has found that miR-22-3p bore tumor-suppressive functions in non-small cell lung cancer^36^. Also, it has been proved in retinoblastoma that miR-22-3p targeted ENO1 to inhibit proliferation^37^. Our study firstly validated in LUAD that cric-ENO1 sponged miR-22-3p to upregulate ENO1 expression. Rescue assays indicated that circ-ENO1 promoted glucolysis and tumor progression of LUAD through miR-22-3p/ENO1. Finally, in vivo assays further validated that circ-ENO1 promoted tumor growth and metastasis in LUAD.

In summary, our study was the first to reveal the circRNA-regulated glycolysis in LUAD, disclosing that circRNA-ENO1 promoted proliferation and EMT in LUAD through upregulating its host gene ENO1, providing circ-ENO1 as a new potential biological marker in lung cancer.

## Supplementary information

Supplementary Figure 1.

Supplementary Figure legend
