# Supplementary figures and images for: CircRNA-ENO1 promoted glycolysis and tumor progression in lung adenocarcinoma through upregulating its host gene ENO1
Source: Cell Death Dis. 2019 Nov 25;10(12):885. doi: 10.1038/s41419-019-2127-7 (PMC6877563; doi:10.1038/s41419-019-2127-7)

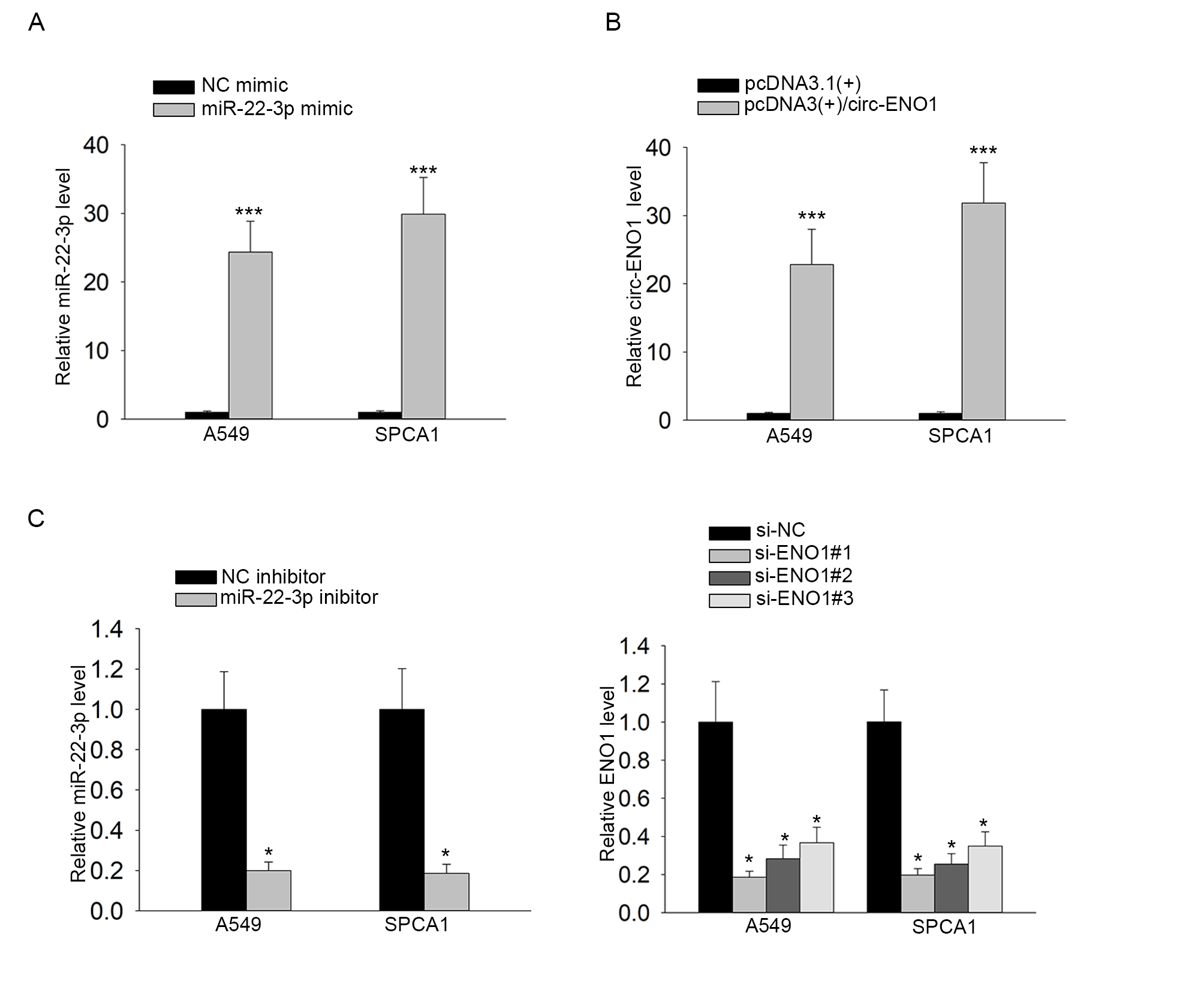

Supplement: Supplementary file 1 — Supplementary Figure 1. [file 41419_2019_2127_MOESM1_ESM.tif]
