# Supplementary material for: CircRNA-ENO1 promoted glycolysis and tumor progression in lung adenocarcinoma through upregulating its host gene ENO1
Source: Cell Death Dis. 2019 Nov 25;10(12):885. doi: 10.1038/s41419-019-2127-7 (PMC6877563; doi:10.1038/s41419-019-2127-7)
Supplement: Supplementary file 2 — Supplementary Figure legend [file 41419_2019_2127_MOESM2_ESM.docx]

**Supplementary Figure 1.** (A) Upregulation of miR-22-3p in A549 and SPCA1 cells was induced by transfection of miR-22-3p mimic. (B) Overexpression of circ-ENO1 was identified with RT-qPCR. (C) Knockdown of miR-22-3p and ENO1 in A549 and SPCA1 cells was determined by RT-qPCR. ^*^*p* < 0.05, ^***^*p* < 0.001.
